# Paediatric use of emergency medical services in India: A retrospective cohort study of one million children

**DOI:** 10.7189/jogh.12.04080

**Published:** 2022-10-16

**Authors:** Jennifer A Newberry, Srinivasa J Rao, Loretta Matheson, Ashri S Anurudran, Peter Acker, Gary L Darmstadt, SV Mahadevan, GV Ramana Rao, Matthew Strehlow

**Affiliations:** 1 Department of Emergency Medicine, Stanford University School of Medicine, Stanford, California, USA; 2 Department of Pediatrics, Stanford University School of Medicine, Stanford, California, USA; 3 GVK Emergency Management and Research Institute, Telangana, India

## Abstract

**Background:**

Millions of children in low- and middle-income countries (LMICs) experience illness or trauma amenable to emergency medical interventions, but local resources are not sufficient to treat them. Emergency medical services (EMS), including ambulance transport, bridge the gap between local services and higher-level hospital care, and data collected by EMS could be used to elucidate patterns of paediatric health care need and use. Here we conducted a retrospective observational study of patterns of paediatric use of EMS services by children who used EMS in India, a leader in maternal and child EMS development, to inform public health needs and system interventions to improve EMS effectiveness.

**Methods:**

We analysed three years (2013-2015) of data from patients <18 years of age from a large prehospital EMS system in India, including 1 101 970 prehospital care records across 11 states and a union territory.

**Results:**

Overall, 38.3% of calls were for girls (n = 422 370), 40.5% were for adolescents (n = 445 753), 65.9% were from rural areas (n = 726 154), and most families were from a socially disadvantaged caste or lower economic status (n = 834 973, 75.8%). The most common chief complaints were fever (n = 247 594, 22.5%), trauma (n = 231 533, 21.0%), and respiratory difficulty (n = 161 120, 14.6%). However, transport patterns, including patient sex and age and type of destination hospital, varied by state, as did data collection.

**Conclusions:**

EMS in India widely transports children with symptoms of the leading causes of child mortality and provides access to higher levels of care for geographically and socioeconomically vulnerable populations, including care for critically ill neonates, mental health and burn care for girls, and trauma care for adolescents. EMS in India is an important mechanism for overcoming transport and cost as barriers to access, and for reducing the urban-rural gap found across causes of child mortality. Further standardisation of data collection will provide the foundation for assessing disparities and identifying targets for quality improvement of paediatric care.

Millions of children in low- and middle-income countries (LMICs) die unnecessarily from causes such as preterm birth, pneumonia, diarrheal diseases, birth asphyxia and trauma despite substantial efforts to reduce paediatric morbidity and mortality [1]. India has some of the world’s highest rates of paediatric mortality and more than double the number of under-5 deaths of all other LMICs, except for Nigeria [2]. The leading causes of neonatal death in India are prematurity, infections, birth asphyxia, and birth trauma, whereas half of post-neonatal under-5 deaths in India are due to pneumonia and diarrhoea [3]. Most of these causes are amenable to emergency medical interventions, such as those provided by emergency medical services (EMS).

EMS is a critical but under-recognised component of global public health infrastructure. EMS provides emergency medical treatment and stabilisation, decreases time to definitive care, and connects patients to the broader health care system. Currently, research on EMS in LMICs is sparse; a literature review identified studies from 16 countries, with little to no information available regarding their paediatric services [4]. However, two obstacles impeding the development of EMS systems were highlighted: insufficient funding for the implementation and ongoing needs of ambulance services; and widespread poverty, which limits the ability of patients to pay for care or transport. In India, low-cost systems (INR₹878 or US$15 per EMS response) supported by public-private partnerships have overcome these challenges to provide free-of-charge EMS to anyone – including rural and economically disadvantaged populations – connecting them to primary, secondary, and tertiary care facilities and allowing for rapid life-saving interventions [5-7].

Many EMS systems routinely collect data needed for clinical care that can also provide insights into the frequency and distribution of conditions which are severe or urgent enough to require transport, and the demographics of those with these conditions. These data sets are a rich, but underutilised resource for understanding patterns of public health needs, potentially allowing specific resources to be directed to areas where they will have the largest effect on outcomes, in accordance with World Health Organization (WHO) goals [8].

In this study, we aimed to describe paediatric EMS utilisation patterns across India. We hypothesized that patients from rural and lower socioeconomic groups would predominate and that patient chief complaints would reflect common causes of childhood mortality.

## METHODS

### Study design and setting

Since 2005, Gunupati Venkata Krishna Emergency Management and Research Institute (GVK EMRI) has provided adults and children in India access to free prehospital emergency medical care and ambulance transport. Working with state governments, GVK EMRI has expanded to 15 states and a union territory – a catchment area of over 750 million people. State-based call centres receive calls and coordinate ambulance dispatch 24 hours a day, 7 days a week. The specific training for emergency medical technicians (EMTs), which includes 45 days of foundational training, orientation to evidence-based guidelines, and annual refresher trainings, has been described elsewhere [9]. Standard protocol directs ambulances to the nearest government health care facility, but EMT discretion and patient preference may influence the facility ultimately chosen. Trained EMTs treat patients aboard ambulances equipped for emergency response and transport.

We conducted a retrospective analysis of paediatric dispatch records from January 1, 2013, through December 31, 2015, across 11 states and a union territory (**Table 1**). This time period was chosen based on availability and consistency of data gathering processes across states. Trained emergency response officers answer calls at each state’s central call centre and dispatch the nearest available ambulance using GPS. At the time of dispatch, computerised data collection systems are used to record only essential demographic data, including age, sex, social status, economic status, and incident area; no outcome data are recorded.

**Table 1:** Demographics of paediatric patients using emergency medical services in India (2013-2015)

	Total		Sex				Age group								Age in years	
		**Female**		**Male**		**Neonate**		**Infant**		**Child**		**Adolescent**			
						**<1 mo**		**1 mo-1 y**		**>1 y-<10 y**		**10 y-<18 y**			
**n (%)**		**n (% of n)**				**n (% of n)**								**Median (IQR)**	
**Total**	1 101 970	(100.0)	422 370	(38.3)	622 589	(56.5)	159 049	(14.4)	126 101	(11.4)	314 182	(28.5)	445 753	(40.5)	7	(1-14)
**Andhra Pradesh**	80 129	(7.3)	33 076	(41.3)	47 053	(58.7)	12 408	(15.5)	12 982	(16.2)	24 740	(30.9)	29 998	(37.4)	6	(0.9-13)
**Assam**	150 260	(13.6)	46 565	(31.0)	70 795	(47.1)	3201	(2.1)	11 049	(7.4)	43 667	(29.1)	59 455	(39.6)	10	(3-14)
**Gujarat**	141 949	(12.9)	52 992	(37.3)	80 865	(57.0)	7571	(5.3)	23 512	(16.6)	44 585	(31.4)	58 238	(41.0)	7	(2-14)
**Himachal Pradesh**	61 332	(5.6)	24 968	(40.7)	30 870	(50.3)	4581	(7.5)	8364	(13.6)	19 825	(32.3)	23 069	(37.6)	7	(1-13)
**Karnataka**	110 118	(10.0)	46 157	(41.9)	63 961	(58.1)	31 640	(28.7)	15 082	(13.7)	29 895	(27.1)	33 501	(30.4)	3	(0.08-12)
**Meghalaya**	6067	(0.6)	2782	(45.9)	3116	(51.4)	1797	(29.6)	606	(10.0)	1739	(28.7)	1757	(29.0)	3	(0.08-11)
**Rajasthan**	104 903	(9.5)	25 760	(24.6)	68 787	(65.6)	14 127	(13.5)	3788	(3.6)	42 660	(40.7)	34 035	(32.4)	6	(2-12)
**Tamil Nadu**	82 791	(7.5)	35 711	(43.1)	47 080	(56.9)	54 724	(66.1)	11 099	(13.4)	7864	(9.5)	9104	(11.0)	0.08	(0.08-1)
**Telangana**	73 665	(6.7)	32 608	(44.3)	41 057	(55.7)	11 096	(15.1)	9663	(13.1)	22 333	(30.3)	30 573	(41.5)	7	(1-14)
Union Territory*	4652	(0.4)	2170	(46.6)	2482	(53.4)	261	(5.6)	1329	(28.6)	–	–	3062	(65.8)	13	(0.9-15)
**Uttar Pradesh**	255 414	(23.2)	106 850	(41.8)	148 564	(58.2)	13 906	(5.4)	24 389	(9.5)	68 588	(26.9)	148 531	(58.2)	11	(5-15)
**Uttarakhand**	30 690	(2.8)	12 731	(41.5)	17 959	(58.5)	3737	(12.2)	4237	(13.8)	8286	(27.0)	14 430	(47.0)	8	(1-14)

IQR – interquartile range

Missing – sex 5.2%, age 5.2%.

*Union Territory – Dadra and Nagar Haveli and Daman and Diu.

### Study population

All calls for patients <18 years were eligible for inclusion. Exclusion criteria were dispatches for pregnancy, with no EMT-patient contact, or with replicate case identifiers. Pregnancy-related cases were excluded because (1) some states provided non-emergent pregnant or postpartum transport services that could not be distinguished from emergent cases, and (2) pregnancy-related calls were sometimes erroneously classified as paediatric based on the delivery of a newborn, rather than the initial call from the mother. Cases without EMT contact included hoax calls and dispatches where the patient was missing or refused services. When calls regarding multiple patients (eg, food poisoning, mass casualty incidents) led to replicate case identifiers, we included only the first instance.

### Measurements

Age was recorded in years, whole or fractional. Age “0” either referred to a newborn infant or indicated missing data. Where age was “0” and the original chief complaint indicated a neonatal condition (eg, neonatal tetanus), we assumed an age of <1 month and re-coded it with the more specific value 0.083 (1/12 of a year). If age was “0”, sex was missing, and there was no indication that the condition was neonatal, the age was re-coded as missing; otherwise, it remained as “0”.

Social status was defined by using the proxy variable of caste with routinely used categories [10-12]. Individuals identifying as “other caste” are the least economically and socially disadvantaged; “scheduled caste”, “backward caste”, “other backward caste”, and “scheduled tribe” are the most disadvantaged. Economic status was defined by whether the family reported having a ration card for subsidised food. Eligibility for a ration card includes living below the poverty line (INR₹972/mo, or about US$16.20/mo) or having certain characteristics such as homelessness [13,14]. Location was categorised by dispatch officers as either urban, rural, or tribal. The majority of states only used the urban and rural categories and included tribal within the rural category. Consequently, we combined rural and tribal location categories when used. The definition of “urban” was not universal but generally encompassed well-known large metropolitan areas.

Dispatchers categorised calls into emergency types and subtypes that varied across states. We mapped varying emergency types and subtypes to 15 major chief complaint categories (Table S1 in the **Online Supplementary Document****)**. Categories were defined by consensus between experienced global health emergency medicine researchers at Stanford and research partners at GVK EMRI. Final categories are clinically meaningful and similar to those used by the National EMS Information System (NEMSIS) in the United States [15].

We categorised hospitals as government or non-government. Non-government hospitals included private, non-governmental non-profit, trust-supported, and private hospitals that receive partial funding from the national government. We further categorised government hospitals by care capacity level: primary, secondary, or tertiary (multispecialty or teaching). This categorisation by capacity is not available for non-government hospitals. Because this was a free-text field, certain entries were ultimately undecipherable into the above categories (n = 28 885, 2.6%).

### Analysis

Data management and analysis were done in SAS Enterprise Guide, version 8.2 (SAS Institute Inc., Cary, NC, USA). We report descriptive statistics for key variables, with median and interquartile ranges for non-normally distributed data. We compared continuous variables using *t* tests (or Wilcoxon rank sum tests when appropriate) and categorical variables using χ^2^ tests. A *P* < 0.05 was considered statistically significant. This study was approved by GVK EMRI and the Stanford University Institutional Review Board (#46662). Given that this study utilizes a retrospective observational cohort analysis approach, patients were not involved in the design, conduct, choice of outcome measures, recruitment, or dissemination of this study. Paediatric patient records remained anonymised while their data was analysed retrospectively for this study.

## RESULTS

**Figure 1:**
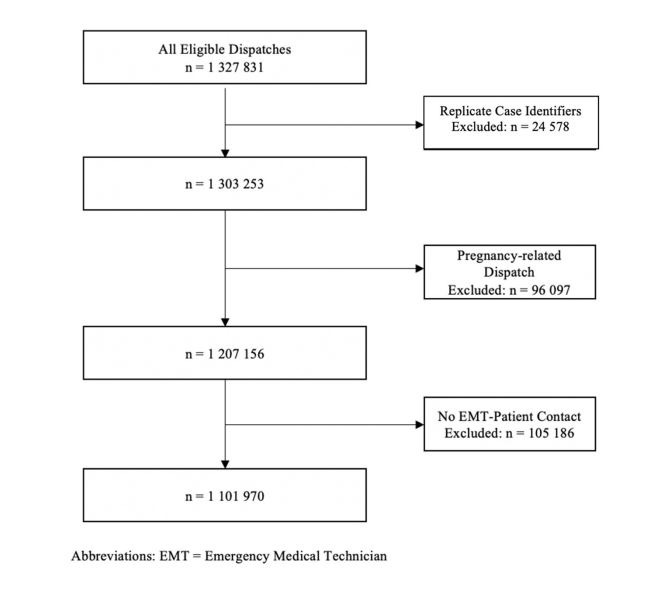
Cohort map.

From 2013 to 2015, there were 1 327 831 emergency medical dispatch records for patients <18 years of age (**Figure 1**). After removing records meeting the exclusion criteria, we analysed 1 101 970 records including 12 247 patients who were dead on arrival (1.1%), 24 414 who received some aid but were not transported (2.2%), and 1 065 309 who received emergency medical transport (96.7%).

Overall, the proportion of female patients was significantly lower than that of the general population (38.3% vs 47.6%, *P* < 0.001) (**Table 1**) [16]. The median age for the entire cohort was 7 years (interquartile range (IQR) = 1-14). Among patients with a reported location (n = 942 430), 77.1% (n = 726 154) were in rural or tribal areas (**Table 2**). This is true across all states – children were predominantly from rural or tribal locations (*P* < 0.0001). Of patients with a reported economic status, 89.0% (n = 840 919) had a ration card, an indication of poor economic status. This trend also was true for all states (*P* < 0.0001) except for Karnataka (*P* = 0.4). Of patients with recorded economic and social status (n = 861 582), 96.9% (n = 834 973) had either a ration card or were from a disadvantaged caste; 75.5% (n = 650 831) had both a ration card and were from a disadvantaged caste (**Table 2**). Patient demographics varied notably by state. For example, at least two-thirds of patients were from rural or tribal areas in nearly all states, but >80% of patients from Himachal Pradesh, Rajasthan, and Uttar Pradesh were from rural or tribal areas.

**Table 2:** Family location, economic status, and social status of patients using paediatric emergency medical services in India (2013-2015)

	Total		Location				Economic status				Economic and social status							
		**Urban**		**Rural or tribal**		**Ration card**		**No ration card**		**Ration card, disadvantaged caste**		**Ration card, other caste**		**No ration card, disadvantaged caste**		**No ration card, other caste**	
**n (%)**		**n (% of n)**				**n (% of n)**				**n (% of n)**							
**Total**	1 101 970	(100.0)	216 276	(19.6)	726 154	(65.9)	840 919	(76.3)	104 446	(9.5)	650 831	(59.1)	122 769	(11.1)	61 373	(5.6)	26 609	(2.4)
**Andhra Pradesh**	80 129	(7.3)	25 116	(31.3)	54 510	(68.0)	70 114	(98.7)	1015	(1.3)	69 954	(87.3)	9127	(11.4)	389	(0.5)	143	(0.2)
Assam*	150 260	(13.6)	–	–	–	–	116 158	(77.3)	1059	(0.7)	53 509	(35.6)	6734	(4.5)	78	(0.1)	10	(0.01)
**Gujarat**	141 949	(12.9)	44 437	(31.3)	97 512	(68.7)	132 799	(93.6)	1047	(0.7)	113 074	(79.7)	19 674	(13.9)	285	(0.2)	126	(0.1)
**Himachal Pradesh**	61 332	(5.6)	4023	(6.6)	51 241	(83.5)	49 192	(80.2)	12 140	(19.8)	22 285	(36.3)	17 275	(28.2)	3104	(5.1)	1624	(2.6)
**Karnataka**	110 118	(10.0)	37 478	(34.0)	72 640	(66.0)	54 912	(49.9)	55 160	(50.1)	38 283	(34.8)	16 547	(15.0)	34 884	(31.7)	19 147	(17.4)
**Meghalaya**	6067	(0.6)	1876	(30.9)	4191	(69.1)	5883	(97.0)	15	(0.2)	5555	(91.6)	160	(2.6)	6	(0.1)	5	(0.1)
**Rajasthan†**	104 903	(9.5)	12 636	(12.0)	92 267	(88.0)	–	–	–	–	–	–	–	–	–	–	–	–
**Tamil Nadu**	82 791	(7.5)	21 420	(25.9)	58 706	(70.9)	81 529	(98.5)	1262	(1.5)	80 082	(96.7)	1331	(1.6)	71	(0.1)	3	(0.004)
**Telangana**	73 665	(6.7)	25 711	(34.9)	47 910	(65.0)	72 513	(98.4)	1151	(1.6)	67 134	(91.1)	5348	(7.3)	309	(0.4)	146	(0.2)
**Union Territory‡**	4652	(0.4)	542	(11.7)	4110	(88.3)	4437	(95.4)	215	(4.6)	4296	(92.3)	139	(3.0)	42	(0.9)	7	(0.2)
**Uttar Pradesh**	255 414	(23.2)	36 142	(14.2)	219 272	(85.8)	218 064	(85.4)	27 010	(10.6)	187 301	(73.3)	29 488	(11.5)	21 637	(8.5)	2961	(1.2)
**Uttarakhand**	30 690	(2.8)	6895	(22.5)	23 795	(77.5)	26 318	(85.8)	4372	(14.2)	9358	(30.3)	16 946	(55.2)	568	(1.9)	2437	(7.9)

Missing – location 14.5%, economic status 14.2%, social status 21.8%.

*Location unavailable for Assam.

†Economic status and social status unavailable for Rajasthan.

‡Union territory – Dadra and Nagar Haveli and Daman and Diu.

Across states, the most common chief complaints were fever, trauma, and respiratory difficulty (**Table 3** and Table S2 in the **Online Supplementary Document** includes chief complaints by state). The frequency of chief complaints varied considerably by age (**Table 3**). Among neonates (age <1 month), almost half of the calls were for respiratory difficulty (47.9%); this remained the most common chief complaint for infants (<1 year of age) (31.2%), along with fever (28.6%). As children aged, fever remained common, but trauma emerged increasingly, becoming the most common chief complaint among adolescents (29.2%). Among calls with a reported sex, males represented 70.0% (n = 148 597) of trauma calls (**Table 3**). Females represented 63.4% (n = 4697) of patients with mental health complaints and nearly half with abdominal pain (48.0%, n = 51 983), toxicology (49.1%, n = 15 910), and burns (48.7%, n = 10 291), despite representing 40.4% (n = 422 370) of the cohort with a reported sex. In burns this disparity is even more noticeable when age is taken into account: females under the age of 10 years represented 43.3% (n = 3359) of burn patients in that age range, but adolescent girls aged 10 and older represented 61.4% (n = 4894) of burn patients of that age. Overall, there were significant differences in gender representation for all chief complaints (diabetes-related *P* < 0.05; cardiac *P* < 0.01; stroke and toxicology *P* < 0.005; all others *P* < 0.0001).

**Table 3:** Chief complaints of paediatric patients using emergency medical services across India, by sex and age (2013-2015)

	Total		Sex				Age group								Age	
		**Female**		**Male**		**Neonate**		**Infant**		**Child**		**Adolescent**			
						**<1 mo**		**1 mo-1 y**		**>1 y-<10 y**		**10 y-<18 y**			
**n (% of n)**		**n (% of n)**				**n (% of n)**								**Median (IQR)**	
**Total**	1 101 970	(100.0)	422 370	(38.3)	622 589	(56.5)	159 049	(14.4)	125 875	(12.3)	314 180	(27.7)	445 753	(40.5)	7	(1-14)
**Chief complaints, most frequent**																
Fever	247 594	(22.5)	99 075	(40.0)	141 550	(57.2)	7316	(3.0)	35 969	(15.6)	95 253	(37.4)	102 096	(41.2)	8	(2-13)
Trauma	231 533	(21.0)	63 665	(27.5)	148 597	(64.2)	1047	(0.5)	6246	(3.3)	74 863	(31.7)	130 106	(56.2)	12	(6-15)
Respiratory difficulty	161 120	(14.6)	65 612	(40.7)	91 679	(56.9)	76 212	(47.3)	39 323	(24.9)	21 046	(12.6)	20 742	(12.9)	0.9	(0.08-2)
Abdominal pain	112 452	(10.2)	51 083	(46.2)	56 391	(50.1)	1415	(1.3)	8087	(7.8)	26 224	(22.8)	72 648	(64.6)	12	(7-15)
Vomiting/diarrhoea	73 885	(6.7)	30 793	(41.7)	40 955	(55.4)	2761	(3.7)	14 074	(20 9)	27 539	(35.4)	27 374	(37.0)	6	(1-12)
Convulsion	52 169	(4.7)	22 071	(42.3)	29 557	(56.7)	3512	(6.7)	8585	(17.1)	20 590	(38.8)	18 941	(36.3)	6	(2-12)
Other*	86 988	(7.9)	28 770	(33.1)	48 143	(55.3)	32461	(37.3)	6415	(8.6)	19 985	(21.7)	18 110	(20.8)	1	(0.08-9)
**Chief complaints, least frequent**																
Toxicology	34 168	(3.1)	15 910	(46.6)	16 499	(48.3)	152	(0.4)	2166	(7.4)	9876	(27.9)	20 215	(59.2)	12	(5-16)
Neonatal tetanus	30 056	(2.7)	12 798	(42.6)	17 231	(57.3)	30 056	(100.0)	–	–	–	–	–	–	0.08	(0.08)
Burns	27 447	(2.0)	10 291	(45.8)	10 860	(48.4)	100	(0.4)	1578	(7.8)	7469	(32.5)	12 004	(53.5)	11	(5-15)
Cardiac	20 378	(1.8)	8198	(40.2)	8559	(42.0)	683	(3.4)	1823	(9.3)	4422	(21.3)	9829	(48.2)	12	(4-15)
Loss of consciousness	14 937	(1.4)	5720	(38.3)	6774	(45.4)	3181	(21.3)	1155	(8.5)	3303	(21.3)	4855	(32.5)	5	(0.08-13)
Mental health	7632	(0.7)	4697	(61.5)	2709	(35.5)	27	(0.4)	–	–	1433	(18.7)	5838	(76.5)	15	(12-16)
Stroke/paralysis	4611	(0.4)	1951	(42.3)	2152	(46.7)	59	(1.3)	306	(6.9)	1627	(35.0)	2111	(45.8)	10	(5-14)
Diabetes related	1874	(0.2)	779	(41.6)	864	(46.1)	62	(3.3)	148	(8.1)	550	(29.2)	883	(47.1)	10	(5-15)

Missing – chief complaint 0.01%, sex 5.2%, age 5.2%.

*“Other” includes calls where the specific chief complaint is recorded as “other”, the chief complaint is an age category (eg, “neonate”), or the chief complaint did not make clinical sense for age.

Most patients were transported to a government hospital (n = 839 666, 76.2%); of these, most were transported directly to secondary care facilities (n = 510 257, 60.8%) (**Table 4**). Transport patterns varied by state: for example, Uttar Pradesh and Assam were more likely to use primary care facilities than other states. EMS transport patterns also varied significantly by age (*P* < 0.001); neonates were the most likely to be transported to a secondary or tertiary care facility, whereas adolescents were mostly transported to secondary care facilities and much less to tertiary care.

**Table 4:** Receiving hospital of paediatric patients using emergency medical services across India, by sex, age, and chief complaint (2013-2015)

	Total		Government								Non-government* all levels	
		**Primary care level**		**Secondary care level**		**Tertiary care level**		**Unknown level**			
**n (%)**		**n (% of n)**								**n (% of n)**	
**Total**	1 101 970	(100.0)	88 338	(8.0)	510 257	(46.3)	127 008	(11.5)	114 063	(10.4)	148 783	(13.5)
**Sex**												
Female	422 370	(38.3)	35 925	(8.5)	208 364	(49.3)	52 284	(12.4)	48 287	(11.4)	57 760	(13.7)
Male	622 589	(56.5)	52 366	(8.4)	301 499	(48.4)	74 678	(12.0)	65 572	(10.5)	90 825	(14.6)
**Age group**												
Neonate, <1m	159 049	(14.4)	9148	(5.8)	43 816	(27.5)	38 933	(24.5)	31 424	(19.8)	23 232	(14.6)
Infant, 1m-1y	135 121	(12.3)	6976	(5.2)	50 609	(37.5)	19 941	(14.8)	15 412	(11.4)	26 971	(20.0)
Child, >1y-<10y	305 162	(27.7)	26 132	(8.6)	161 977	(53.1)	30 734	(10.1)	28 766	(9.4)	47 753	(15.6)
Adolescent, 10-<18y	445 753	(40.5)	46 035	(10.3)	253 463	(56.9)	37 354	(8.4)	38 258	(8.6)	50 630	(11.4)
**Chief complaint†**												
Fever	247 594	(22.5)	27 394	(11.1)	132 320	(53.4)	19 186	(7.7)	22 847	(9.2)	27 814	(11.2)
Trauma	231 533	(21.0)	16 435	(7.1)	114 669	(49.5)	19 055	(8.2)	19 555	(8.4)	29 395	(12.7)
Respiratory	161 120	(14.6)	10 162	(6.3)	53 878	(33.4)	33 125	(20.6)	21 936	(13.6)	30 804	(19.1)
Abdominal pain	112 452	(10.2)	13 991	(12.4)	62 832	(55.9)	8368	(7.4)	9428	(8.4)	10 399	(9.2)
Vomiting/diarrhoea	73 885	(6.7)	6330	(8.6)	38 277	(51.8)	5451	(7.4)	5703	(7.7)	13 973	(18.9)
Convulsion	52 169	(4.7)	1901	(3.6)	20 113	(38.6)	8265	(15.8)	5480	(10.5)	13 708	(26.3)
Other	86 988	(7.9)	4710	(5.4)	31 589	(36.3)	11 794	(13.6)	11 320	(13.0)	6189	(7.1)
**State**												
Andhra Pradesh	80 129	(7.3)	2445	(3.1)	31 667	(39.5)	21 913	(27.3)	995	(1.2)	19 111	(23.9)
Assam	150 260	(13.6)	21 534	(14.3)	77 190	(51.4)	11 299	(7.5)	475	(0.3)	5315	(3.5)
Gujarat	141 949	(12.9)	1248	(0.9)	69 426	(48.9)	5433	(3.8)	14 074	(9.9)	38 721	(27.3)
Himachal Pradesh	61 332	(5.6)	2753	(4.5)	13 473	(22.0)	8114	(13.2)	28 618	(46.7)	325	(0.5)
Karnataka	110 118	(10.0)	10 277	(9.3)	28 812	(26.2)	–	–	234	(0.2)	66 952	(60.8)
Meghalaya	6067	(0.6)	476	(7.8)	2750	(45.3)	–	–	1355	(22.3)	1142	(18.8)
Rajasthan	104 903	(9.5)	4988	(4.8)	50 982	(48.6)	4215	(4.0)	3701	(3.5)	1029	(1.0)
Tamil Nadu	82 791	(7.5)	733	(0.9)	10 247	(12.4)	32 485	(39.2)	37 238	(45.0)	1163	(1.4)
Telangana	73 665	(6.7)	2204	(3.0)	35 698	(48.5)	20 335	(27.6)	553	(0.8)	12 582	(17.1)
Union Territory‡	4652	(0.4)	389	(8.4)	4049	(87.0)	–	–	–	–	92	(2.0)
Uttar Pradesh	255 414	(23.2)	39 227	(15.4)	168 848	(66.1)	20 532	(8.0)	26 779	(10.5)	25	(0.01)
Uttarakhand	30 690	(2.8)	2064	(6.7)	17 115	(55.8)	2682	(8.7)	41	(0.1)	2326	(7.6)

Missing or Undeciphered – receiving facility type 10.3%, sex 5.2%, age 5.2%.

*Non-government includes private, non-governmental non-profit, trust supported, and government supported.

†Showing only most common chief complaints with total >4% of all calls.

‡Union Territory – Dadra and Nagar Haveli and Daman and Diu. There are no tertiary care facilities in Dadra and Nagar Haveli and Daman and Diu.

## DISCUSSION

With over 1 million records, this study of paediatric EMS is the largest reported to date and provides important insights into population-level characteristics and transport patterns for children in India. The centralised, state-based EMS system connected a mostly rural, economically and socially disadvantaged paediatric population to the public health care system. It cared for children experiencing symptoms and signs of the leading causes of neonatal, infant, child, and adolescent mortality and morbidity (such as respiratory difficulty, serious infections, and trauma) and mostly transported them to higher levels of care, where paediatric and critical care are more likely to be available. This was particularly true for neonates, infants, and those with respiratory complaints, although this varied across states, reflecting the different ways in which states may integrate EMS into their health systems.

### Connecting children to care

In India, public health care is organised across three levels of increasing care capacity: primary care (most common, limited capacity, mostly single-physician coverage), secondary care, and tertiary care (least common, at least one facility per district) [15]. The most common chief complaints seen in this study – fever, respiratory difficulty, and trauma – may require diagnostic and treatment capabilities that are unavailable at more prevalent, closer primary care facilities. National guidelines indicate that secondary care facilities at a minimum should be staffed by a paediatrician, have the ability to run basic laboratory tests and perform x-rays, and have access to intravenous medications for paediatric patients [17]. In line with this, the majority (60.8%) of patients in this study who were taken to a government facility were transported to secondary care facilities. Further, nearly half of calls for neonates with respiratory complaints were preferentially transported to secondary or tertiary care facilities, which are more likely to have specialised neonatal care units with resources for critically ill neonates, including respiratory support. Although the general guidelines are to transport patients to the nearest facility, we found that factors such as young age and need for advanced care were associated with transport to higher levels of care, which are most often further away. EMS thus appears to overcome transport as a barrier to access, which has been identified as one of the most common obstacles to care [18].

Improving timely diagnosis and treatment and increasing care-seeking are two critical steps for reducing child mortality [19,20]. The high demand for paediatric trauma-related emergency services, accounting for 21.0% of all chief complaints, represents an important opportunity to improve outcomes, particularly for adolescents. WHO has advocated for EMS prehospital trauma systems as a critical first step in the chain of survival for paediatric traumatic injuries [8,21]. Prehospital care both reduces morbidity and mortality from trauma and is cost-effective [22]. Condition-specific studies of India’s EMS system indicate it decreases time to care by initiating critical treatment en route, including intravenous fluids and supplemental oxygen [5,7,23-25]. In addition, our study’s identification of the most common chief complaints among transported patients can help governments and emerging EMS agencies direct resources toward care for these conditions, further reducing time to care and maximising the system’s impact on health outcomes. However, funding is often focused on primary care with little focus on implementation of emergency care systems for the treatment of time-sensitive conditions [26]. Our data underscore the importance of investment in the prehospital emergency care arena.

### Reaching the most vulnerable families

We found that EMS consistently reached children from economically and socially vulnerable homes – 96.9% were from disadvantaged backgrounds. Children from the poorest households have twice the mortality rate as those from the richest households [27]. In India, private hospitals in urban environments commonly operate small fleets of fee-for-service ambulances, providing more affluent households access to emergency care. The robust use of the free-of-charge EMS system by disadvantaged and rural populations suggests that it helps to broaden access for these groups.

Of reported locations, 77.1% were rural. Infant and child mortality occurs predominantly in rural areas of India (91%), with only 17% of all deaths occurring in hospitals prior to widespread EMS availability [3]. Children in rural areas are less likely to receive care from a health care provider for pneumonia or oral rehydration solution for diarrhoea [19]. Further, road-traffic injuries, while more frequent in urban areas, tend to be of greater severity in rural areas [28]. Thus, EMS has the potential to address the urban-rural gap found across causes of paediatric mortality [4].

Our analysis indicated that EMS served 147 males for every 100 females in a context where India’s under-18 sex ratio is 110 males to 100 females [16]. Two chief complaints were notable for being overrepresented by a particular sex: trauma (boys accounted for 70.0% of calls) and mental health (girls accounted for 63.4% of calls). The high proportion of boys among EMS trauma calls is consistent with current literature [21,29]. Given the prevalent burden and infrequent help-seeking practices for mental health in India among young women and girls [30], it is intriguing that female paediatric patients used EMS for mental as well as physical health services. Access to emergency care for burns associated with gender-based violence and disparity may be an important component in multisectoral programs to promote gender equality [31]. These patterns and their relation to broader sex-specific health outcomes and gender-related care-seeking patterns should be further investigated, including with regard to the role of EMS.

Finally, our study demonstrates the valuable role an EMS system can play as a data collection and dissemination hub across wide geographic areas. By analysing information already collected by EMS, we provide insights that can guide targeted training endeavours, resource distribution, and quality improvement efforts both within the ambulance service and within state-level health systems. The data can also show population-level disparities and drive targeted efforts to reduce barriers for vulnerable patients.

### Limitations and implications for data quality improvement

This study’s primary limitation is data quality and a lack of standardised dispatch practices across states. States do not currently use the same mutually exclusive categories for emergency types. A core set of demographic data should be used across all states and coded in a standardised manner [8], for example by using WHO’s Standardized Clinical Form, which was released in January 2020 to support the standardisation of data collection for medical and trauma emergencies at the facility level [32]. Standardising chief complaints would improve generalisability, create opportunity for population-level health monitoring, and make coding calls easier for time-constrained dispatch officers. More accurate descriptions of symptoms and categorisation of chief complaints could strengthen epidemiological research on paediatric EMS utilisation and would facilitate future research on the impact of EMS on time-to-care and associated morbidity and mortality from the leading causes of paediatric death. Practice changes recently undertaken by the EMS system in this study, such as allowing age to be entered in months and years, thereby clearly distinguishing neonates and infants, will improve the precision and accuracy of reporting. In addition, the WHO data set for injury is a minimum standardised data set that could be used across EMS agencies [33] as a step toward Sustainable Development Goal 17.18, to “enhance capacity-building support to developing countries” by increasing the availability of high-quality, timely, and reliable data disaggregated by characteristics relevant in national contexts.

## CONCLUSION

Although paediatric deaths remain unacceptably high in LMICs, India’s EMS system is connecting children with signs and symptoms of the most common causes of mortality and morbidity to the country’s health care services. The large representation of high-risk, historically low-utilising populations – neonatal, adolescent, rural, and socioeconomically disadvantaged – suggests that India’s EMS system offers an effective means of increasing health care access for those at highest risk. This study also uncovers sex-specific differences in EMS utilisation, and the factors leading to these disparate patterns must be evaluated further. Finally, this study highlights the value of EMS systems as data sources that could be used to drive quality improvement as well as to identify and reduce disparities in health care. This vital knowledge offers an opportunity to further explore paediatric health burdens and work toward providing equitable access to care for vulnerable children in India and other LMICs.

## Additional material


Online Supplementary Document
