# Supplementary material for: Paediatric use of emergency medical services in India: A retrospective cohort study of one million children
Source: J Glob Health. 2022 Oct 16;12:04080. doi: 10.7189/jogh.12.04080 (PMC9569422; doi:10.7189/jogh.12.04080)
Supplement: Online Supplementary Document [file jogh-12-04080-s001.pdf]

ONLINE SUPPLEMENTARY DOCUMENT

Title: Pediatric use of emergency medical services in India: A retrospective observational cohort study of one million children

Authors: Jennifer A Newberry, Srinivasa J Rao, Loretta Matheson, Ashri S Anurudran, Peter Acker, Gary L Darmstadt, SV Mahadevan, GV Ramana Rao, Matthew Strehlow

Table S1. Chief complaint categories and original call subtypes

Chief Complaint Category	Original Call Subtype	
Abdominal pain	Acute abdomen (non-traumatic)	
	Abdominal pain	
	Colic/pain	
	Colic	
Burns		
	Acid burns/corrosives/hot powders	Explosives
	Electrical burns	Exposure to flames/fire
	Electrocution (lightening)	Exposure to hot liquids/gases
	Electrocution (non-lightening)	Forest fire
	Electrocution (current shock)	Liquefied Petroleum Gas
	Explosive fire	Radiation burns
Cardiac	Blood pressure problem (high and low blood pressure)	Dysrhythmias
	Cardiac	Hypotension (low blood pressure)
	Chest pain (acute coronary syndrome)	Malignant hypertension (elevated blood pressure)
	Chest pain	
Convulsions	Convulsions	
	Febrile convulsions	
	Seizure/convulsions/fits	
	Status epilepsy	
Diabetes-related	Diabetic complications	
	Diabetic problem (sugar problem)	
	Hyperglycemic/hypoglycemic coma	
	Hypoglycemia in known cases	
Fever		Illness
	Fever	Meningitis/encephalitis
	Fever (all types)	Septicemia/infections
	Fevers, Cerebral malaria, Typhoid, Hemorrhagic fevers, etc. Hyperpyrexia	Septicemia
Loss of consciousness	Coma (altered level of consciousness)	
	Fainting	
	Syncope	
	Unconscious	
Mental health	Acute manic reaction	Suicide
	Depression	Suicide attempt (all types)
	Mental problem	Suicide (hanging/slashing wrist/cut throat)
	Panic attacks	
Neonatal tetanus	Neonatal tetanus	

Pregnancy	Abnormal presentations<br>Abortions<br>Antenatal check-up<br>Bleeding in pregnancy (after or before delivery)<br>Delivery at scene<br>Delivery in ambulance<br>Eclampsia<br>Convulsions in pregnancy	Fetal loss<br>In labor<br>Janani Shishu Suraksha Karyajaram scheme<br>Medical conditions complicating pregnancy<br>Post-caesarian labor<br>Precious pregnancy<br>Pregnancy/child birth/miscarriage/labor pains
Respiratory	Anaphylaxis/croup<br>Anaphylaxis<br>Acute respiratory distress syndrome (ARDS)<br>ARDS/Shortness of breath<br>Asthma<br>Breathing problem<br>Chocking	Foreign body aspiration obstruction<br>Inhalation injuries<br>Insects (anaphylaxis)<br>Not breathing (cardiac arrest/respiratory arrest)<br>Pneumonias and influenza<br>Rapid breathing
Stroke/Paralysis	Paralysis<br>Stroke (paralysis)<br>Transient attacks (mini-strokes)	
Toxicology	Allergic reaction - Drugs/Others<br>Chemical poisoning (Cyanide/others)<br>Drugs<br>Drunkenness<br>Explosives - Poisonous gases<br>Herbal poisoning<br>Nuclear Materials	Organophosphate poisoning<br>Overdose - Drugs<br>Petroleum Products<br>Poisoning (Insecticides/Pesticides etc.)<br>Poisonous gases (Carbon monoxide/Methane)<br>Scorpion bites<br>Snake bites
Trauma	2 Wheeler accidents<br>4 Wheeler accidents<br>Accident - Domestic<br>Accident - Domestic/Non-vehicular<br>Accident - Vehicular<br>Accident<br>Amputations<br>Animal Bite/Insect Bite<br>Animal Bite<br>Assault (Including Sexual Assault) / Violence<br>Assault (Including Sexual Assault)<br>Auto (3 Wheeler accidents)<br>Bleeding injuries<br>Crush injuries (wall collapse/slab collapse/stuck between objects)<br>Crush injuries<br>Cut by sharp object<br>Disasters due to natural element	Industrial Accident (Including Fire)<br>Manmade Disasters<br>Mass casualty incident (MCI)<br>Multiple Victims MCI<br>Multiple Vehicular Incident (MVC)<br>MVC - Pedestrian (Run Over/Hit & Run)<br>MVC-Fixed object (Divider)<br>MVC-MVC<br>MVC-Non traffic inside a parked vehicle/Skidding<br>MVC-Thrown off from Vehicle<br>Natural Disasters<br>Near drowning (Suicidal/accidents)<br>Non-motorized vehicle accidents (Bullock cart/Bicycle/Rickshaw/Push cart)<br>Penetrating injury by sharp object<br>Railroad Train/Other Object<br>Sexual<br>Skid & fall

	Dog bite<br>Drowning<br>Extrication process<br>Extrication lasting >20 minutes<br>Fall from height- Building/Electrical poles/Bridges/Staircase/Tree/Ladder<br>Fall victim<br>Fracture<br>Gunshot<br>Head injuries<br>Hit by blunt object<br>Hit by object/animal<br>Hypothermia<br>Inaccessible incident (Bore Well/Well)	Smothering/Hanging/Throttling/Lynching<br>Stab/ Gunshot wound<br>Structural collapse (House/Roof/Wall)<br>Train - Other Object<br>Train accident<br>Trapped in machinery<br>Trauma<br>Traumatic arrest<br>Unarmed fight/Brawl<br>Vehicle -Fall From Height etc.<br>Vehicle role over/Skid<br>Vehicular trauma<br>Wild animals
Vomiting and diarrhea	Dehydration<br>Diarrhea/Dehydration<br>Food poisoning<br>Gastritis/Acidity/Blood Vomiting	Gastroenteritis<br>GI/Dehydration<br>Rectal bleeding<br>Vomiting
Other	Acute renal failure/Retention of urine<br>Back pain (Nontraumatic/Nonrecent trauma)<br>ENT Bleed<br>Exposure to Cold<br>Headache<br>Heat/Sun Stroke	Jaundice<br>Massive pulmonary embolism<br>Steven Johnson Syndrome<br>Tension pneumothorax<br>Water contamination<br>Others

Table S1. Most-frequent chief complaints of pediatric patients using emergency medical services across India, by state (2013-2015)

	Total		Fever		Trauma		Respiratory Difficulty		Abdominal Pain		Vomiting/Diarrhea		Convulsion		Other†	
	N (%)		n (% of N)													
**Total**	1 101 970	(100.0)	247 594	(22.5)	231 533	(21.0)	161 120	(14.6)	112 452	(10.2)	73 885	(6.7)	52 169	(4.7)	86 988	(7.9)
**Andhra Pradesh**	80 129	(7.3)	14 383	(17.9)	16 429	(20.3)	14 411	(18.0)	5 952	(7.4)	5 904	(7.4)	6 682	(8.3)	4 416	(5.5)
**Assam**	150 260	(13.6)	42 656	(28.4)	25 183	(16.8)	8 990	(6.0)	20 334	(13.5)	20 908	(13.9)	600	(0.4)	15 710	(10.5)
**Gujarat**	141 949	(12.9)	27 089	(19.1)	40 559	(28.6)	14 012	(9.9)	9 678	(6.8)	15 558	(11.0)	14 285	(10.1)	4 854	(3.4)
**Himachal Pradesh**	61 332	(5.6)	18 044	(29.4)	9 914	(16.2)	4 180	(6.8)	8 375	(13.7)	4 157	(6.8)	1 036	(1.7)	12 415	(20.2)
**Karnataka**	110 118	(10.0)	17 415	(15.8)	13 452	(12.2)	37 240	(33.8)	7 157	(6.5)	11 278	(10.2)	7 346	(6.7)	3 247	(2.9)
**Meghalaya**	6 067	(0.6)	932	(15.4)	1 610	(26.5)	445	(7.3)	889	(14.7)	640	(10.5)	234	(3.9)	210	(3.5)
**Rajasthan**	104 903	(9.5)	24 927	(23.8)	25 017	(23.8)	5 114	(4.9)	6 899	(6.6)	2 273	(2.2)	625	(0.6)	31 487	(30.0)
**Tamil Nadu**	82 791	(7.5)	5 577	(6.7)	2 308	(2.8)	32 477	(39.2)	1 795	(2.2)	2 585	(3.1)	5 199	(6.3)	9 673	(11.7)
**Telangana**	73 665	(6.7)	13 155	(17.9)	13 188	(17.9)	11 803	(16.0)	7 348	(10.0)	5 277	(7.2)	6 865	(9.3)	3 993	(5.4)
**Union Territory**	4 652	(0.4)	1 212	(26.1)	903	(19.4)	250	(5.4)	616	(13.2)	1 021	(21.9)	76	(1.6)	193	(4.1)
**Uttar Pradesh**	255 414	(23.2)	76 978	(30.1)	73 504	(28.8)	26 883	(10.5)	37 417	(14.6)	3 186	(1.2)	7 798	(3.1)	764	(0.3)
**Uttarakhand**	30 690	(2.8)	5 226	(17.0)	9 646	(31.4)	5 315	(17.3)	5 992	(19.5)	1 098	(3.6)	1 423	(4.6)	26	(0.1)

Union Territory: Dadra and Nagar Haveli and Daman and Diu.
Missing: Chief Complaint 0.01%.
†'Other' includes calls where the specific chief complaint is 'Other', the chief complaint is an age category (e.g. 'Neonate'), of the chief complaint did not make clinical sense for age.

Table S2. Least-frequent chief complaints of pediatric patients using emergency medical services across India, by state (2013-2015)

	Total		Toxicology		Neonatal Tetanus		Burns		Cardiac		Loss of Conscious-ness		Mental Health		Stroke/ Paralysis		Diabetes Related	
	N (%)		n (% of N)															
**Total**	1 101 970	(100.0)	34 168	(3.1)	30 056	(2.7)	22 447	(2.0)	20 378	(1.8)	14 937	(1.4)	7 632	(0.7)	4 611	(0.4)	1 874	(0.2)
**Andhra Pradesh**	80 129	(7.3)	2 657	(3.3)	3 196	(4.0)	1 307	(1.6)	1 384	(1.7)	272	(0.3)	2 646	(3.3)	378	(0.5)	244	(0.3)
**Assam**	150 260	(13.6)	4 165	(2.8)	15	(0.01)	2 576	(1.7)	3 704	(2.5)	4 382	(2.9)	288	(0.2)	510	(0.3)	238	(0.2)
**Gujarat**	141 949	(12.9)	4 895	(3.4)	1 611	(1.1)	2 897	(2.0)	2 855	(2.0)	2 723	(1.9)	310	(0.2)	220	(0.2)	400	(0.3)
**Himachal Pradesh**	61 332	(5.6)	1 042	(1.7)	40	(0.1)	761	(1.2)	999	(1.6)	180	(0.3)	27	(0.04)	101	(0.2)	61	(0.1)
**Karnataka**	110 118	(10.0)	5 787	(5.3)	984	(0.9)	1 647	(1.5)	3 133	(2.8)	679	(0.6)	174	(0.2)	306	(0.3)	269	(0.2)
**Meghalaya**	6 067	(0.6)	48	(0.8)		(0.0)	60	(1.0)	356	(5.9)	462	(7.6)	20	(0.3)	121	(2.0)	40	(0.7)
**Rajasthan**	104 903	(9.5)	1 768	(1.7)	1 540	(1.5)	1 775	(1.7)	1 873	(1.8)	1 246	(1.2)	117	(0.1)	139	(0.1)	95	(0.1)
**Tamil Nadu**	82 791	(7.5)	1 346	(1.6)	19 288	(23.3)	502	(0.6)	646	(0.8)	1 251	(1.5)	12	(0.01)	64	(0.1)	56	(0.1)
**Telangana**	73 665	(6.7)	2 532	(3.4)	2 746	(3.7)	1 020	(1.4)	1 211	(1.6)	269	(0.4)	3 753	(5.1)	277	(0.4)	189	(0.3)
**Union Territory**	4 652	(0.4)	203	(4.4)	22	(0.5)	50	(1.1)	51	(1.1)	47	(1.0)	4	(0.1)	1	(0.02)	3	(0.1)
**Uttar Pradesh**	255 414	(23.2)	9 032	(3.5)	581	(0.2)	9 176	(3.6)	3 904	(1.5)	3 287	(1.3)	206	(0.1)	2 453	(1.0)	235	(0.1)
**Uttarakhand**	30 690	(2.8)	693	(2.3)	33	(0.1)	676	(2.2)	262	(0.9)	139	(0.5)	75	(0.2)	41	(0.1)	44	(0.1)

Union Territory: Dadra and Nagar Haveli and Daman and Diu.
Missing: Chief Complaint 0.01%.
